# Differences in cytokines expression between Vero cells and IPEC-J2 cells infected with porcine epidemic diarrhea virus

**DOI:** 10.3389/fmicb.2022.1002349

**Published:** 2022-11-10

**Authors:** Chen Yuan, Lidan Sun, Ligong Chen, Limin Li, Zuojun Yao, Yawen Wang, Haiyong Guo, Tanqing Li, Qinye Song

**Affiliations:** ^1^Veterinary Biological Technology Innovation Center of Hebei Province, College of Veterinary Medicine, Hebei Agricultural University, Baoding, China; ^2^School of Life Science, Jilin Normal University, Siping, China

**Keywords:** PEDV, Vero cells, IPEC-J2 cells, cytokines, difference

## Abstract

Porcine epidemic diarrhea virus (PEDV) primarily infects suckling piglets and causes severe economic losses to the swine industry. Cytokines, as part of the innate immune response, are important in PEDV infection. The cytokines secreted by cell infection models *in vitro* might reflect true response to viral infection of target cells *in vivo*. Vero cells and IPEC-J2 are commonly used as an *in vitro* model to investigate PEDV infection. However, it is not clear which type of cells is more beneficial to the study of PEDV. In our study, firstly, Vero cells and IPEC-J2 were successfully infected with PEDV virulent strains (HBQY2016) and attenuated vaccine strains (CV777) and were capable of supporting virus replication and progeny release. Moreover, cytokine differences expression by Vero cells and IPEC-J2 cells infected with two PEDV strains were analyzed. Compared with IPEC-J2 cells, only the mRNA levels of TGF-β, MIP-1β and MCP-1 were detected in Vero cells. ELISA assay indicated that compared to the control group, the PEDV-infected group had significantly induced expression levels of IL-1β, MIP-1β, MCP-1, IL-8, and CXCL10 in IPEC-J2 cells, while only secretion level of IL-1β, MIP-1β and IL-8 in Vero cells were higher in PEDV infected group. Finally, cytokines change of piglets infected PEDV-HBQY2016 strains were detected by cDNA microarray, and similar to those of IPEC-J2 cells infected PEDV. Collectively, these data determined that the IPEC-J2 could be more suitable used as a cell model for studying PEDV infection *in vitro* compared with Vero cells, based on the close approximation of cytokine expression profile to *in vivo* target cells.

## Introduction

Porcine enteric coronaviruses mainly include porcine epidemic diarrhea virus (PEDV), porcine deltacoronavirus (PDCoV), porcine transmissible gastroenteritis virus (TGEV) and swine acute diarrhea syndrome coronavirus (SADS-CoV), which have posed a catastrophic threat to the pig industry and caused serious economic losses worldwide (Jung and Saif, 2015; Jung et al., 2016; Zhang et al., 2022). Of the above four viruses, PEDV has been recognized as the most destructive pathogen in recent years, with a mortality rate of up to 100% in neonatal pigs under 1 week of age (Song and Park, 2012; Sun et al., 2012). PEDV was first reported in the United Kingdom in the early 1970s and has since become endemic in some areas (Pensaert and de Bouck, 1978). In 2010, there was a large outbreak of porcine epidemic diarrhea (PED) in China (Li et al., 2012), and in 2013, PEDV also emerged in the United States and spread rapidly, posing significant economic loss to the pork industry (Stevenson et al., 2013; Chen et al., 2014). With the emergence of highly virulent PEDV strains, studies on PEDV have increased. Currently, variant PEDV strains are involved in the continuing epidemic. However, the classical strain-derived vaccines provide low or failed to protect against the PEDV variants. And the safety, low or no cross-immune protection between various strains and titers of virus culture *in vitro* about live attenuated vaccines are the challenges of PEDV vaccine development (Niu and Wang, 2022).

Porcine epidemic diarrhea virus is an enveloped single-stranded RNA (ssRNA) virus that targets the small intestine and replicates mainly in intestinal epithelial cells (Pensaert and de Bouck, 1978; Lee, 2016). In the process of virus proliferation, PEDV could rapidly destroy and break away from the intestinal physical structure, further resulting in villus atrophy of the small intestine and even rupture of the mucosal barrier, finally leading to imbalance of intestinal osmotic pressure, dehydration, watery diarrhea and even death of piglets (Sun et al., 2012; Niu et al., 2021; Yuan et al., 2021). In the process of PEDV infection, the innate immune response, as the first line of defense, could protect the host from viral infection and control viral infection (Du et al., 2019; Koonpaew et al., 2019).

Cytokines, as part of the innate immune response, are important in viral infection. Based on the structure and function of the cytokines, they could be broadly categorized into interferons, interleukins, the chemokine family, the tumor necrosis factor family and so on (Chauhan et al., 2021). Different types of cytokines could regulate cellular activities, which include-cell to cell communication, differentiation, proliferation and immune responses (Chauhan et al., 2021). It has been reported that the cytokines secreted during a viral infection help to modulate immune responses and maintaining immune homeostasis (Chen Y. M. et al., 2020). For example, IL-6 and IL-8 are important cytokines involved in the immune response and mediating the inflammatory response (Chauhan et al., 2021; Zhang et al., 2022). In addition, cytokines secreted by cell infection models *in vitro* might represent a true response to viral infection of target cells *in vivo.* Therefore, cell infection model is often used *in vitro* experiments to reveal the interaction mechanism between pathogen infection and host immunity.

Cell lines that permit for PEDV replication and stands for intestinal immune responses might contribute to further studies about PEDV (Chen J. et al., 2020). Therefore, it is of great significance to determine proper cell lines for PEDV research. Vero cells and IPEC-J2 are commonly used as an *in vitro* model to investigate PEDV infection (Lin et al., 2017; Chen et al., 2021). However, it is not clear which type of cells is more beneficial to the study of PEDV. The growing evidence demonstrate that cytokines play an important role in PEDV infection and could analyze the immune status of piglets infected with PEDV (Jung et al., 2018; Yu et al., 2019; Chen Y. M. et al., 2020). However, the difference of the cytokines *in vitro* or *in vivo* is not examined thoroughly and warrant further investigation.

In the present study, we first analyzed the levels of partial cytokines secreted by IPEC-J2 and Vero cells infected with the PEDV virulent strain and attenuated strain, respectively. Furthermore, the expression levels of cytokines in ileum tissue of piglets infected with PEDV virulent strain were detected by cDNA microarray. By comparing the results of *in vitro* and *in vivo* experiments, it was proved that the cytokine profile secreted by IPEC-J2 cells infected with PEDV was basically consistent with that of pig ileum tissue, which suggested that IPEC-J2 cells could be an ideal model for studying PEDV infection *in vitro*, based on the close approximation of cytokine expression profile to *in vivo* target cells.

## Materials and methods

### Cells and virus

African green monkey kidney (Vero-81) cells were grown and maintained in Dulbecco’s Modified Eagle’s Medium (DMEM) supplemented with heat-inactivated fetal bovine serum (FBS) at 37°C with 5% CO_2_. IPEC-J2 (kindly provided by Dr. Huanrong Li, Beijing University of Agriculture, Beijing, China) was maintained in DMEM/F12 supplemented with 10% FBS (Gibco). PEDV-CV777 strains (Gen Bank Accession No. KT323979) and PEDV-HBQY2016 strains (Gen Bank Accession No. MH244927) was maintained at the College of Veterinary Medicine, Hebei Agricultural University, Baoding.

### Reagents and antibodies

Trizol reagent, Prime Script^™^ II 1st strand cDNA Synthesis Kit and SYBR Green qPCR Kit were purchased from Takara Biomedical Technology (Beijing) Co., Ltd. LPS were purchased from Solarbio Science and Technology (Beijing) Co., Ltd. FITC-conjugated goat anti-mouse secondary antibody was purchased from Invitrogen (Waltham, MA, United States). Enzyme-Linked Immunosorbent Assay (ELISA) kits were purchased from Dongge Biological (Beijing) Co., Ltd. The anti-PEDV-N antibody was prepared in our laboratory.

### Tissue culture infectious dose (TCID_50_) assay

The Vero cells or IPEC-J2 cells were infected with two different strains of PEDV (HBQY2016 or CV777). At 72 h post infection (hpi), the culture including cells and supernatant were subjected to three rounds of freezing–thawing to release viruses. Viral titer of PEDV HBQY2016 and CV777 strains were determined by TCID_50_. Vero cells or IPEC-J2 were seeded into 96-well plates at 1.6 × 10^4^ cells/well in a volume of 200 μl per well and cultured for 90% confluence. The cell monolayers were extensively washed with PBS. Serial 10-fold dilutions of the PEDV supernatant were made, and 100 μl of each dilution were inoculated to the 96-well plate. Eight repetitions were performed for each dilution. Then the plates were incubated at 37° Cunder the condition of 5% CO_2_. After 3 days, the cell monolayers were performed immunofluorescence assay. The fluorescence signal was checked under a Zeiss inverted fluorescence microscope. The TCID_50_ of the PEDV was calculated based on the Reed-Muench method.

### RNA extraction and RT-PCR

The Vero cells or IPEC-J2 cells were infected with two PEDV strains (HBQY2016 or CV777) at a multiplicity of infection (MOI) of 0.1 for different times at 37°C with 5% CO_2_. Then the cells were collected for the cytokine detection. The total cultures were collected and subjected to three rounds of freezing–thawing to release viruses. After centrifugation, the supernatants were collected for viral RNA extraction. The cell total RNA or the viral RNA was extracted from PEDV infected or the control Vero cells, IPEC-J2 or ileum samples of PEDV-infected or the control piglets e using the Trizol reagent according to the manufacturer’s protocol, and was then reverse-transcribed into cDNA using oligo (dT) as the primer. Real-time PCR were performed in a Roche Light Cycler 96 real-time PCR system as previously described (Guo et al., 2020). The specific genes of IL-1β, IL-6, TGF-β, MIP-1β, MCP-1, IL-8, and CXCL-10 were amplified from porcine lymph nodes or small intestinal tissue and N gene from PEDV, and cloned into pMD-18 T vector, respectively. After these recombinant plasmids were identified by sequencing, the concentration of these recombinant plasmids was determined by Nano Drop 2000. The constructed recombinant plasmids containing the different target genes were used as standard plasmids for real-time quantitative PCR (qPCR) detection. According to the DNA copy number calculation formula [DNA copy numbers (copies/μl) = cDNA concentration (ng/μl) × 10^−9^ × (6.02 × 10^23^)/DNA length (bp) × 660 g/mol.bp], the copy numbers of these recombinant plasmids were calculated. All these plasmids were stored in −20°C before use. Ten-fold serial of the diluted plasmids were used as templates for qPCR to construct standard curves of various target genes. The copy number of the above genes were calculated based on each standard curve. Specific primers used in this study are shown in Table 1.

**Table 1:** Conditions and primers of PCR to amplify genes of cytokines.

Genes	Pimer sequence(5′ → 3′)	Annealing temperature/°C	Prouducts/bp	GenBank accession no.
IL-1β	Forward:TGTTCTGCATGAGCTTTGTG	55	358	M86725
IL-6	Reverse: TCTGGGTATGGCTTTCCTTAG	54	262	NM_214399
IL-6	Forward:TGGCTACTGCCTTCCCTAC	54	262	NM_214399
MIP-1β	Reverse: TCCTGATTGAACCCAGATTG	58	299	AJ311717
TGF-β	Forward: AGCACAATGATCTGGCCGTT	58.4	354	NM-214198
IL-8	Reverse: GCTGAAAGGTGTGACACGGA	54.2	299	M99367
MIP-1β	Forward:CTCTCCTCCAGCAAGACCA	58	299	AJ311717
PEDV-N	Reverse: GCTCAGTTCAGTTCCAAGTCA	54	316	KM604665
MCP-1	Forward:CTCCTGTGCCTGCTGCT	55	282	X79416
β-actin	Reverse: TTCAAGGCTTCGGAGTTT	61.2	276	U07786

### Immunofluorescence assay

Vero cells or IPEC-J2 grown on coverslips were infected with PEDV, CV777 or HBQY2016 strains, at a MOI of 0.1 for different times at 37°C with 5% CO_2_. Vero cells or IPEC-J2 were fixed with 4% paraformaldehyde for 15 min and permeabilized with 0.1% Triton X-100 at room temperature for 10 min. Then, Vero cells or IPEC-J2 were blocked with 0.4% bovine serum albumin (BSA) for 1 h and incubated with the mouse anti-PEDV-N antibody (1:200) for 1 h at 37°C. The cells were washed with 0.01moL/L phosphate-buffered saline (PBS, pH7.4) three times and incubated with a FITC-conjugated goat anti-mouse secondary antibody (1:500). Coverslips were mounted on the microscope glass slides in a mounting buffer. Fluorescence was visualized using a Zeiss inverted fluorescence microscope after washing three times with PBS.

### Cytokine assays by ELISA

Vero cells or IPEC-J2 cells seeded in 6-well culture plates were incubated with PEDV, CV777 or HBQY2016 strains, at MOI of 0.1 for 1 h. Then the supernatants were discarded. And the cells were maintained in Dulbecco’s Modified Eagle Medium (DMEM; Gibico, NY, United States) at 37°C for 24 h. At the same time, a lipopolysaccharide (LPS) group and a control group were set up. Vero-81 or IPEC-J2 cells in the LPS group and the control group were stimulated with LPS (10 μg/mL) (Solarbio, Beijing, China) and 0.01 mol/l PBS (pH7.4) instead of the virus for the same time, respectively. Then the culture supernatant was collected and cytokines were measured using the ELISA kit: Porcine IL-1β ELISA kit (DG50148P), Porcine IL-6 ELISA kit (DG50031P), Porcine TGF-β ELISA kit (DG50119P), Porcine MIP-1β ELISA kit (DG96433Q), Porcine MCP-1 ELISA kit (DG96436Q), Porcine IL-8 ELISA kit (DG91504Q) and Porcine CXCL-10 ELISA kit (DG96438Q). All ELISA kits were purchased from Dongge Biological (Beijing) Co., Ltd. All experiments were performed in accordance with the manufacturer’s instructions.

### Experimental piglets and infection

Six 21-day-old Yorkshire, Landrace and Large White cross-bred piglets were kept in the Experimental Animal Center in Hebei Agricultural University, China. The swine herd was double negative for PEDV, TGEV and rotavirus (RoV) and their antibodies, respectively. The piglets of similar weight were randomly allocated into two groups (PEDV group and the Control group; *n* = 3 per group). Each piglet in the PEDV group was challenged with PEDV-HBQY2016 strain at a dose of 1.5 × 10^6.6^ TCID_50_/mL and the piglets in the control group were inoculated with an equal volume of DMEM in the same manner. Each group of piglets was housed in a strictly separate room with constant humidity and temperature. All piglets were observed twice a day for 3 dpi. At the end of the experiment, the piglets were euthanized by intravenous injection of pentobarbital sodium (100 mg/kg). Ileum tissues were collected for cytokine expression analysis by cDNA microarray. cDNA microarray testing was performed by Shanghai Biotechnology Co., Ltd. Differentially expressed genes of cytokines were screened by fold-change and Student′s *T*-test. The fold-change ≥ 2 and *value of p* < 0.05 were considered as significant gene differences.

All procedures and experiments performed on the piglets were approved by the Institutional Animal Care and Use Committee of Hebei Agricultural University and followed the National Institutes of Health guidelines for animal experiments’ performance.

### Statistical analysis

All experiments were performed independently three times, and data were expressed as the means ± standard error of the means (SEM). Statistical analyses were performed using student’s *t-*test. The *value of p* < 0.05 was considered significant and the *value of p* < 0.01 was considered highly significant.

## Results

### Replication of PEDV in Vero cells

**Figure 1:**
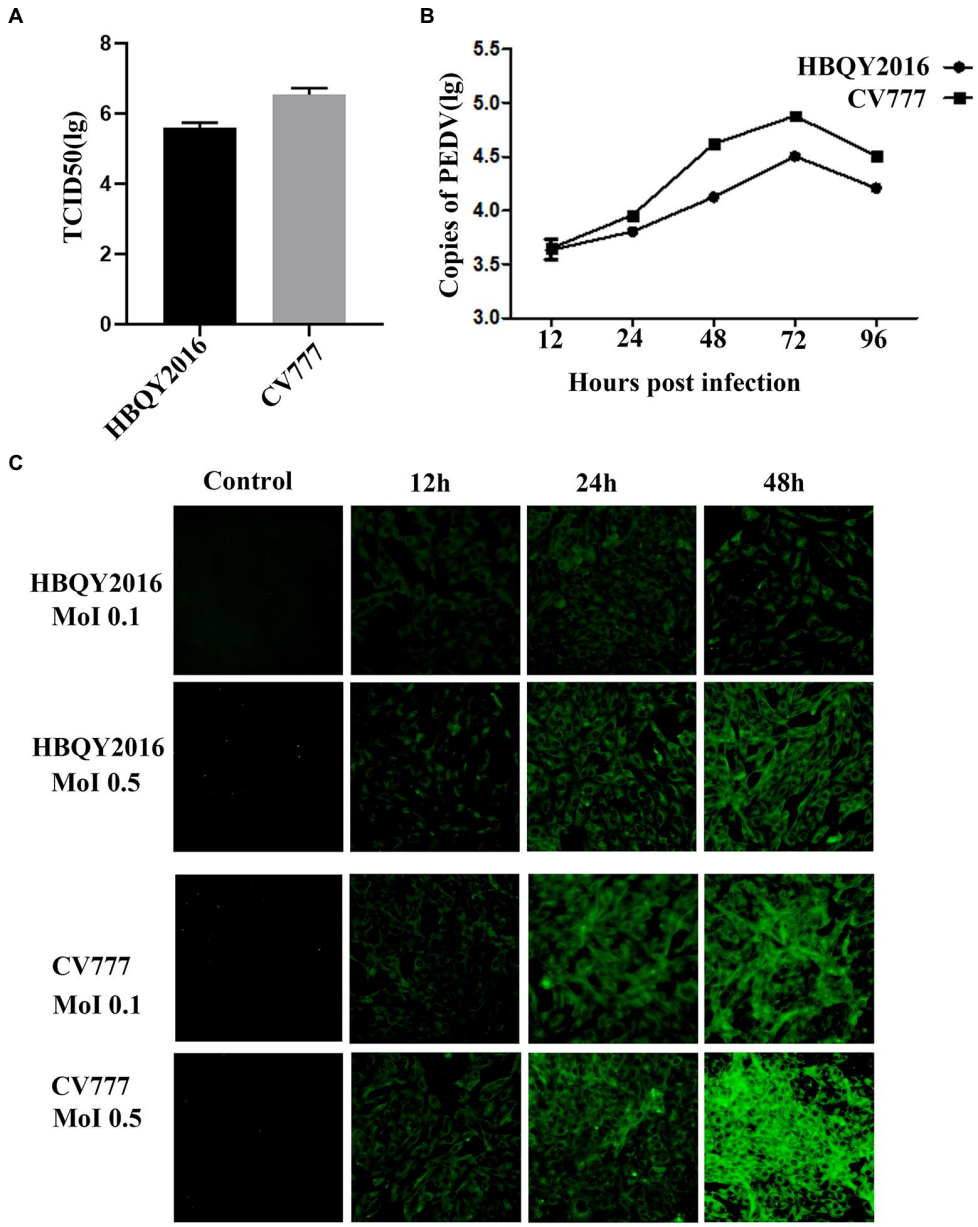
Replication of porcine epidemic diarrhea virus (PEDV) in Vero cells. **(A)** The viral titer of PEDV-HBQY2016 or -CV777 strain was determined with Vero cells with the results expressed as TCID_50_/mL at 72 h post infection (hpi). **(B)** The copies (Log_10_) of PEDV-HBQY2016 or PEDV-CV777 strain in Vero cells at different time points was evaluated by RT-qPCR. **(C)** Immunofluorescence staining of Vero cells infected with PEDV-HBQY2016 or -CV777 strain at different time points.

The Vero cells were infected with PEDV strain HBQY2016 or CV777 and observed for cells with fluorescence under a microscope to calculate virus titers. As shown in Figure 1A, The titers of PEDV HBQY2016 or CV777 strain infected Vero cells were 10^5.6^ TCID_50_/mL and 10^6.5^ TCID_50_/mL, respectively. Then the Vero cells were inoculated with 0.1 MOI of PEDV HBQY2016 or CV777 strain and cultures of the viruses were collected for virus testing by RT-qPCR at 12, 24, 48, 72, and 96 hpi. The results showed that PEDV HBQY2016 and CV777 strains gene copies reached a peak at 72 hpi and declined at 96 hpi. And the total and peak viral titers of PEDV CV777 strain were higher than those of PEDV HBQY2016 strain (Figure 1B). The viral titer was further confirmed by IFA using monoclonal antibodies against PEDV N protein. The IFA showed that the fluorescence signal was slightly visible 12 hpi after inoculation with 0.1 or 0.5 MOI of PEDV HBQY2016 or CV777. The apparent staining was distributed in the cytoplasm and increased rapidly from 24 to 48 hpi. Similarly, the fluorescence intensity of PEDV CV777 strain was higher than that of HBQY2016 strains at different time points with MOI of 0.1 or 0.5 (Figure 1C).

### Replication of PEDV in IPEC-J2 cells

**Figure 2:**
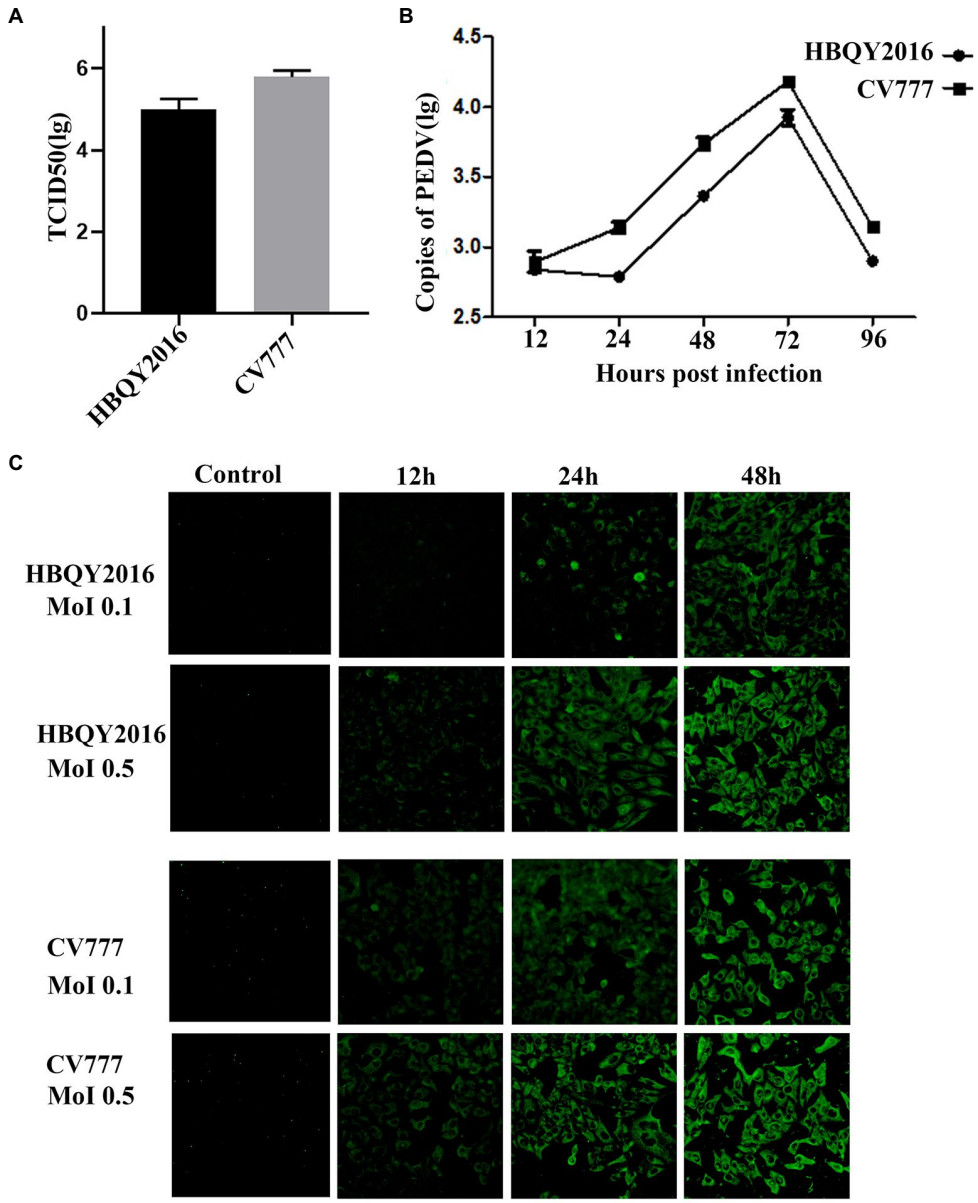
Replication of PEDV in IPEC-J2 cells. **(A)** The viral titer of PEDV-HBQY2016 or -CV777 strain was determined with IPEC-J2 cells with the results expressed as TCID_50_/mL at 72 h post infection. **(B)** The copies (Log10) of PEDV-HBQY2016 or PEDV-CV777 strain in IPEC-J2 cells at different time points was evaluated by RT-qPCR. **(C)** Immunofluorescence staining of IPEC-J2 cells infected with PEDV-HBQY2016 or -CV777 strain at different time points.

The titers of PEDV HBQY2016 or CV777 strains infected IPEC-J2 cells were 10^5.1^ TCID_50_/mL and 10^5.8^ TCID_50_/mL, respectively (Figure 2A). Furthermore, the replication kinetics of HBQY2016 or CV777 strains in IPEC-J2 cells was similar to those of in Vero cells. As shown in Figure 2B, the copy number of viral nucleic acid gradually increased from 12 to 72 hpi, peaked at 72 hpi, and decreased at 96 hpi. Similarly, the fluorescence signal of N protein gradually increased from 12 to 48 hpi for both PEDV-HBQY2016 and PEDV-CV777 strains with MOI of 0.1 or 0.5. And the fluorescence signal of N protein antigen of PEDV-HBQY2016 strain was weaker than that of PEDV-CV777 strains at different time points (Figure 2C).

Taking all these results together, we hold the opinion that that Vero cells and IPEC-J2 cells are susceptible to both PEDV HBQY2016 and CV777 strains. Faster and higher replication kinetics were detected for PEDV CV777 strain in Vero cells and IPEC-J2 cells compared to that of the HBQY2016 strain.

### Cytokine expression in PEDV-infected Vero cells

**Figure 3:**
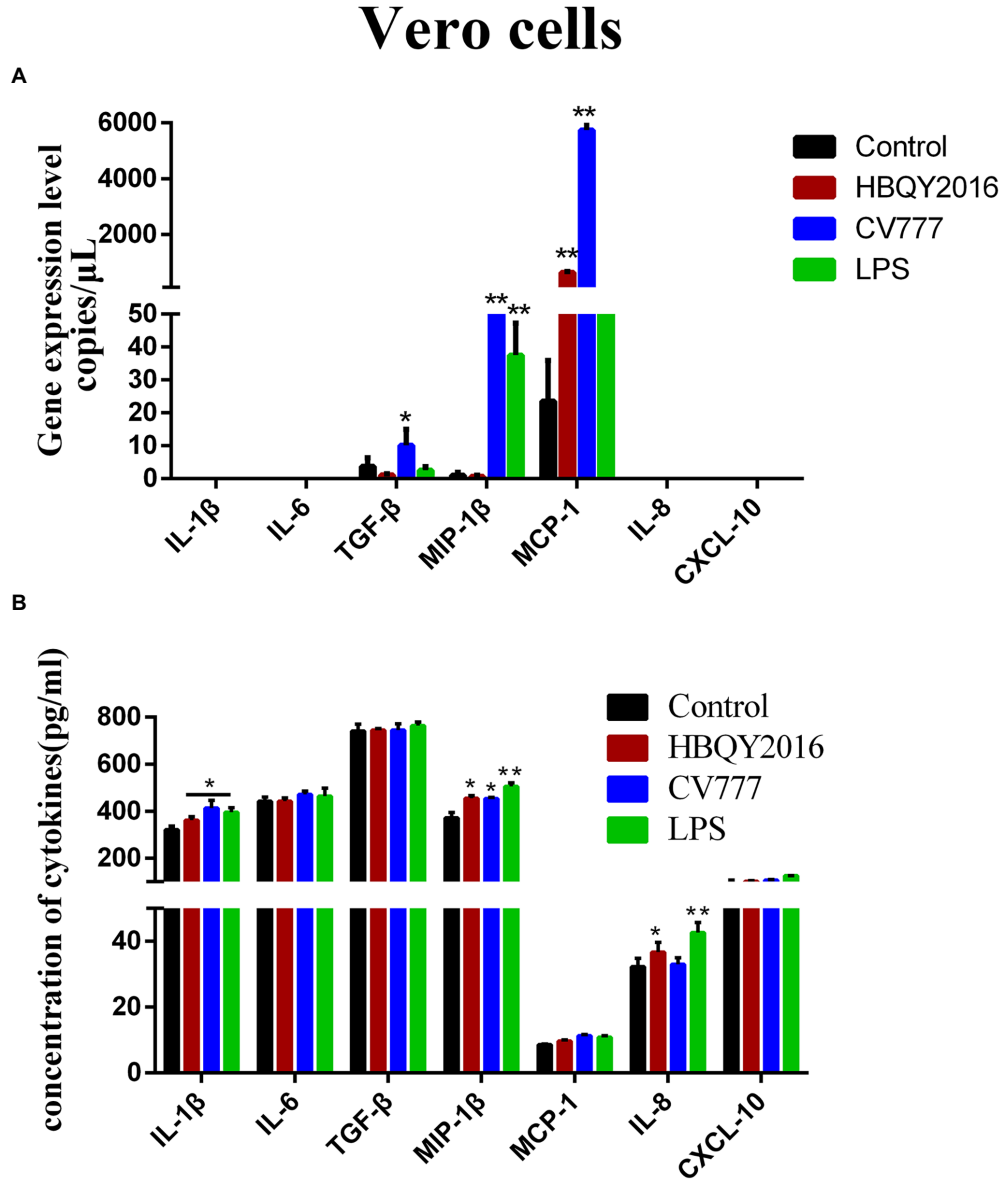
Cytokines expression in Vero cells infected with PEDV. **(A)** Changes of gene copies of IL-1β, IL-6, TGF-β, MIP-1β, MCP-1, IL-8, and CXCL-10 gene expression in Vero cells after PEDV infection. **(B)** Protein concentration of the cytokines secreted by PEDV-infected Vero cells. The supernatant of Vero cells was collected at 24 h after infection with 0.1 MOI PEDV, and the cytokine secretion level was detected by ELISA. ^*^Represents a significant difference relative to the control group (*p* < 0.05), ^**^Represents an extremely significant difference relative to the control group (*p* < 0.01).

As shown in Figure 3A, the mRNA expression levels of MCP-1 were increased in PEDV (PEDV-HBQY2016 and PEDV-CV777 strains) infection group compared with the control group. And the mRNA expression levels of TGF-β and MIP-1β were increased in PEDV-CV777 infected group compared with the control group. However, the mRNA expression levels of other cytokines were particularly low in the PEDV infected and control group, with no significant difference. Next, we examined the difference levels of some important cytokines secreted culture supernatant at 24 h after PEDV infection (Figure 3B). It was found that the secretion levels of IL-1β, MIP-1β, and IL-8 were significantly higher in PEDV infection group than those in the control group except for CV777 induced IL-8 levels. In addition, compared with the control group, the secretion levels of IL-6, TGF-β, MCP-1 and CXCL10 in the PEDV infected group were not significantly different.

### Cytokines expression in PEDV-infected IPEC-J2 cells

**Figure 4:**
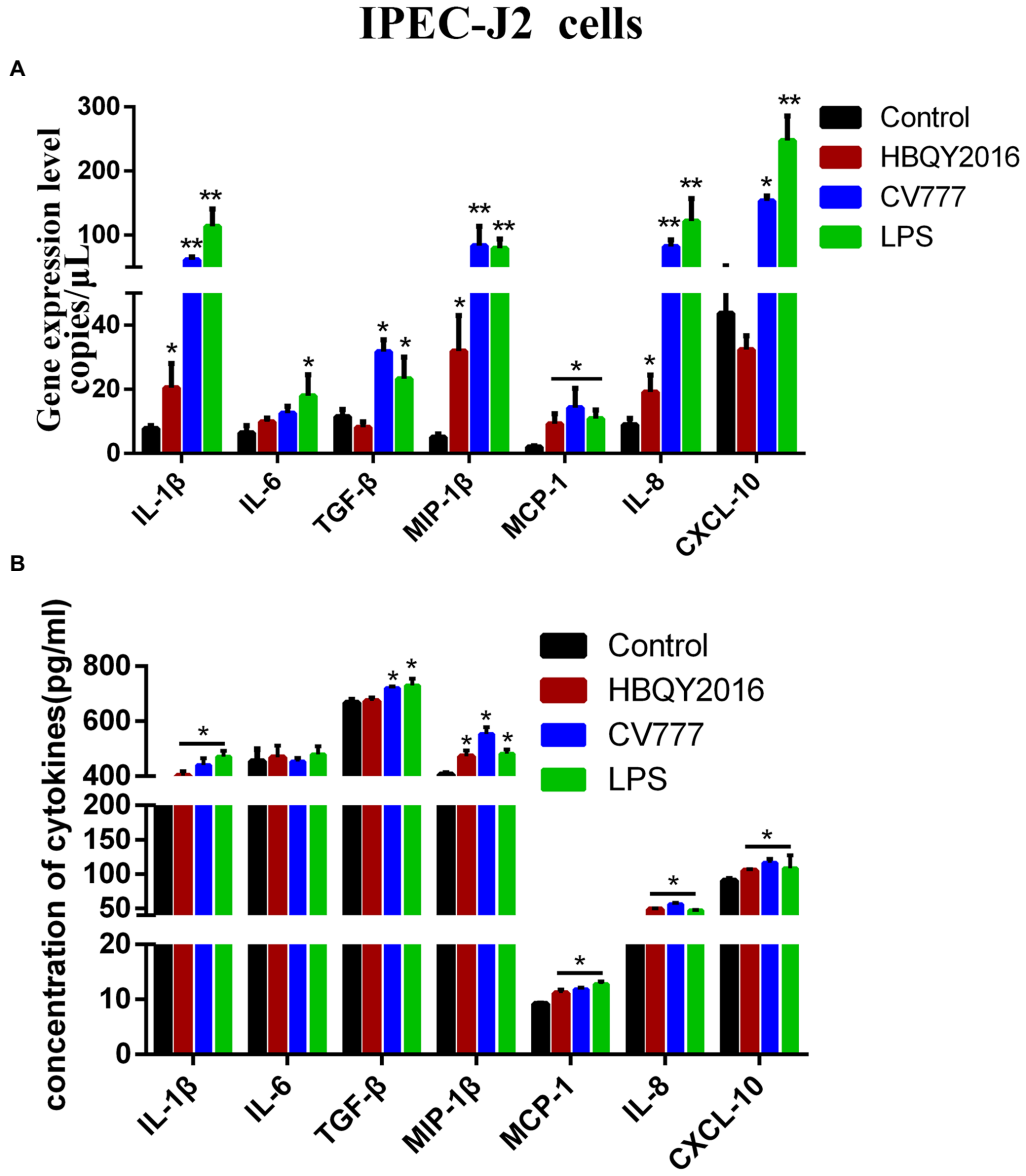
Cytokines expression in IPEC-J2 cells infected with PEDV. **(A)** Changes of gene copies of IL-1β, IL-6, TGF-β, MIP-1β, MCP-1, IL-8, and CXCL-10 gene expression in IPEC-J2 cells after PEDV infection. **(B)** Protein concentration of the cytokines secreted by PEDV-infected IPEC-J2 cells. The supernatant of IPEC-J2 cells was collected at 24 h after infection with 0.1 MOI PEDV, and the cytokine secretion level was detected by ELISA. ^*^Represents a significant difference relative to the control group (*p* < 0.05), ^**^Represents an extremely significant difference relative to the control group (*p* < 0.01).

We also examined the differences in the transcription and protein levels of some cytokines in IPEC-J2 cells at 24 h following PEDV infection using the same experimental design describe above. It was found that the mRNA expressions of IL-1β, MIP-1β, MCP-1, and IL-8 significantly increased after PEDV infection (Figure 4A). The cytokine levels in the cell supernatant measured by ELISA were shown in Figure 4. Compared with the control group, the expressions of IL-1β, MIP-1β, MCP-1, IL-8 and CXCL10 had been significantly induced in PEDV (HBQY2016 and CV777 strains)-infected group. Furthermore, the mRNA and protein levels of TGF-β were also significantly increased in IPEC-J2 cells infected with PEDV-CV777 strains.

### Cytokine expression in piglet ileum induced by oral inoculation with PEDV HBQY2016 strain

**Figure 5:**
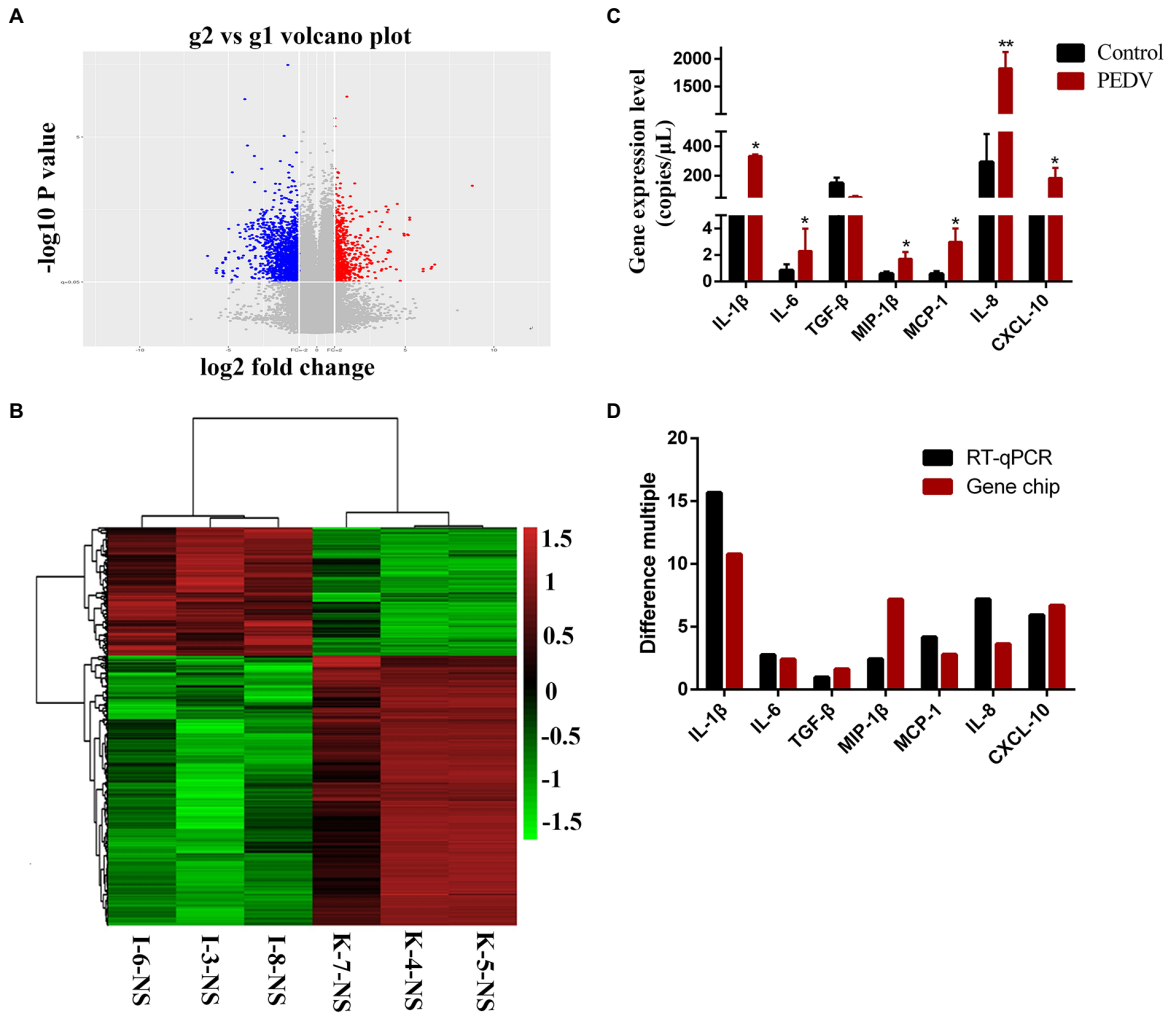
Expression levels of cytokines in ileum of piglets orally inoculated with PEDV. **(A)** The volcano drawing of ileum tissues plot of cDNA microarray. g1 represents ileum tissue of piglets in control group. g2 represents ileum tissue of piglets in PEDV infected group. The ordinate is the -lg (*p*-value) value. The abscissa is log2 (Fold Change). **(B)** Ileum of heat map of cDNA microarray. K, Negative control piglet; I, PEDV-infected piglet. **(C)** The expression levels of the cytokines gene copies in ileum of the PEDV-HBQY2016 infected and the negative control group were quantitatively determined *via* qRT-PCR. **(D)** Comparison of fluorescent quantitative PCR and gene chip results in ileum of piglets. ^*^Represents a significant difference relative to the control group (*p* < 0.05), ^**^Represents an extremely significant difference relative to the control group (*p* < 0.01).

To prove the differential expression profile of cytokines in Vero or IPEC-J2 cells infected with PEDV *in vitro*, we carried out animal challenge experiments. In PEDV group, two piglets developed liquid to semi-liquid diarrhea at day 2 post-infection. Another one pig had semi-liquid diarrhea at day 2 post-infection. Three pigs appeared signs of inappetence at day 2. At necropsy, yellowish watery contents were observed in the small intestine. Before taking samples, PEDV RNA in mucus of the small intestine was detected to confirm that piglets have been infected with PEDV HBQY2016 strain (Supplementary Figure S1). Then total RNA extraction and cDNA microarray detection were performed in ileum of piglets infected with PEDV at 3 days. The differentially expressed genes in the infected group and the control group were classified. The genes with significant difference were screened by fold change and *p*-value obtained by *t* test. As shown in Figure 5A, Volcano plot shows in the same plane that the expression levels of 43,603 genes were changed between the control group (g1) and the infection group (g2), among which 23,348 genes were up-regulated and 20,254 genes were down-regulated. There were 1913 differentially expressed genes (Flod Change ≥ 2 and *p* < 0.05). Next, by comparing the ileum genes of infected piglets with those of the control group using the hierarchical clustering of differential genes, it was found that there were significantly up-regulated and down-regulated genes in the ileum tissues of infected piglets (Figure 5B). Differential expression gene of the cytokines in ileum of piglets infected with PEDV and control group are shown in Table 2. To validate the results of cDNA microarray *in vivo*, the mRNA expression levels of the cytokines in ileum of piglets between the infection group and the control group were quantitatively determined *via* RT-qPCR. After PEDV-HBQY2016 strain infection, the expression levels of IL-1β, IL-6, MIP-1β, MCP-1, IL-8 and CXCL10 were significantly higher than those in in the control group (Figure 5C). Further, by comparing the results of RT-qPCR and cDNA microarray in ileum of piglets, it was found that the change trend of cytokines was similar (Figure 5D). These results indicated that the differential expression of these cytokine genes obtained by RT-qPCR is consistent with gene chip results. Next, the partial cytokine expression profiles *in vitro* (Vero cells and IPEC-J2) and *in vivo* (ileum of piglet) were compared. As shown in Table 3, compared with Vero cells, cytokines secreted by IPEC-J2 cells after PEDV-HBQY2016 infection were similar to those secreted by the ileum *in vivo*, indicating that the IPEC-J2 cells is a better *in vitro* cell model for studying PEDV infection.

**Table 2:** Differential expressed genes of the partial cytokines in ileum of piglets.

Probe name	Gene symbol	Genbank accession	Fold change	Regulation	Description
A_72_P441479	IL-1β	NM_214055	10.78	Up	Sus scrofa interleukin 1, beta 1 (IL1B1), mRNA [NM_214055]
A_72_P177826	IL-6	NM_214399	2.41	Up	Sus scrofa interleukin 6 (interferon, beta 2) (IL6), mRNA [NM_214399]
A_72_P621617	TGF-β	NM_214,198	1.63	Invariant	Sus scrofa transforming growth factor beita (TGF-β), mRNA [NM_214,198]
A_72_P675364	MCP-1	NM_214214	2.80	Up	Sus scrofa chemokine (C-C motif) ligand 2 (CCL2), mRNA [NM_214214]
A_72_P223332	MIP-1β	NM_213779	7.18	Up	Sus scrofa chemokine (C-C motif) ligand 4 (CCL4), mRNA [NM_213779]
A_72_P232367	IL-8/CXCL8	NM_213867	3.63	Up	Sus scrofa chemokine (C-X-C motif) ligand 8 (CXCL8), mRNA [NM_213867]
A_72_P509208	CXCL10	NM_001008691	6.7	Up	Sus scrofa chemokine (C-X-C motif) ligand 10 (CXCL10), mRNA [NM_001008691]

**Table 3:** Comparison of the different in cytokine profiles *in vitro* and *in vivo.*

Detection method	*In vitro*		*In vivo*
	Vero cells	IPEC-J2 cells	Ileum tissue of piglets
qPCR	MCP-1	IL-1β, MIP-1β, MCP-1, IL-8	IL-1β, IL-6, MIP-1β, MCP-1, IL-8, CXCL10
ELISA	IL-1β, MIP-1β, IL-8	IL-1β, MIP-1β, MCP-1, IL-8, CXCL10	
cDNA microarray			IL-1β, IL-6, MIP-1β, MCP-1, IL-8, CXCL10

## Discussion

Since 2010, PEDV variant strains have been widely disseminated in China (Zhao et al., 2017), and many studies have focused on the relationship between host innate immunity and viral infection (Koonpaew et al., 2019; Chen Y. M. et al., 2020). Nowadays, Vero cells and IPECJ2 cells are commonly used in PEDV research (Lin et al., 2017; Chen et al., 2021). However, it has not been reported which kind of cells is more suitable for studying the relationship between PEDV and host innate immunity. In this research, we chose PEDV virulent strains (HBQY2016) or attenuated strains (CV777) as study model and compared the expression differences of some important cytokines *in vivo* and *in vitro* (Vero and IPECJ2 cells) based on PEDV infection to screen better cell models for PEDV studies *in vitro*.

Two different strains of PEDV (HBQY2016 or CV777) were used firstly to detect the susceptibility of Vero cells and IPEC-J2 cells respectively, and they were found to be highly susceptible. The growth curves of PEDV HBQY2016 and CV777 strains in Vero cells were similar to IPEC-J2 cells. Although the gene copy number of the two PEDV strains peaked at 72hpi and decreased at 96 hpi in both IPEC-J2 and Vero cells, the titers of PEDV in Vero cells were higher than those in IPEC-J2 cells. And the virus titer of PEDV-CV777 strains in Vero cells and IPEC-J2 cells was higher than that of PEDV-HBQY2016 strains. These results indicated that PEDV could infect and proliferate on IPEC-J2 cells, which is consistent with the reported results (Annamalai et al., 2015; Lin et al., 2017; Song et al., 2022).

Innate immune response is the host’s first line of defense against the invasion of pathogenic microorganisms. A systematic understanding of the relationship between PEDV infection and innate immunity could provide a new strategy for the prevention and treatment of PED (Zhang and Yoo, 2016). Humoral immunity plays an important role in host resistance to PEDV infection (Langel et al., 2019). We have reported a comprehensive feature of humoral immune response to piglets infected with a virulent PEDV strain (Guo et al., 2020). Similarly, cytokines are essential in antiviral infection and immune regulation (Zhang and Yoo, 2016). It has been reported that PEDV infection could induce inflammatory cytokine responses in intestinal epithelial cells (Lin et al., 2017). In our study, cytokine differences between Vero cells and IPEC-J2 cells infected with different strains of PEDV (HBQY2016 or CV777) were monitored. The results showed that the PEDV had significantly induced secretion of IL-1β, MIP-1β, MCP-1, IL-8 and CXCL10 in IPEC-J2 cells, while only secretion level of IL-1β, MIP-1β and IL-8 in Vero cells were increased. A number of studies have shown that trends of pro-inflammatory factors and chemokines secreted by IPEC-J2 cells infected by PEDV are consistent with the results of this study (Yu et al., 2019; Hu et al., 2021). In addition, cytokines change of piglets infected PEDV-HBQY2016 strains were detected using cDNA microarray and RT-PCR, and similar to those expressed by IPEC-J2 cells infected PEDV. Cytokines are known to be involved in host immune responses. Therefore, it is very important to select an appropriate cell model for *in vitro* studies on the relationship between immune responses and PEDV infection, based on the role of cytokines.

Although PEDV primarily impacts the jejunum and ileum of piglets, interestingly, some researchers reported that there were no significant differences in some important pro-inflammatory factors in jejunum between the control group and PEDV-infected pigs, and suggested that ileum of piglets might be a more sensitive sample type to detect potential cytokine changes (Chen Y. M. et al., 2020). Therefore, in this study, the ileum of infected piglets was selected for cytokine gene expression profiling by cDNA microarray. The expression of pro-inflammatory factors, transformation factors and chemokines induced by PEDV infection in IPEC-J2 cells was similar to that of PEDV-infected piglets *in vivo*, while the expression of the above cytokines in Vero cells was significantly different from that those of PEDV-infected piglets *in vivo*. These results suggest that IPEC-J2 cells are more suitable for *in vitro* infection cell line to study the pathogenesis of PEDV. In addition, IPEC-J2 cells also provide convenience for the study of some intestinal infectious diseases from swine, and are currently recognized as an *in vitro* model for the study of intestinal mucosal immunity (Arce et al., 2010; Zakrzewski et al., 2013).

In present study, there are two reasons for substituting 21-day-old piglets for neonatal suckling piglets in the experiment. One is that neonatal piglets are more susceptible to infection with PEDV due to their naïve and immature immune system, resulting in a high mortality rate. The other is that the natural immune system of 21-day-old piglets is relatively mature, and generally the 21-day-old piglets infected with PEDV will not die, which is conducive to carrying out subsequent experiments. Moreover, viral infections induce both innate and adaptive immunity. Innate immunity provides a first line of immune defense against early viral infection. As a part of innate immunity, cytokines play an important role in early resistance to viral infection. At the same time, the cytokines secreted during a viral infection also involved in the regulation of immune response and the initiation of acquired immune response (Du et al., 2019; Koonpaew et al., 2019; Chen Y. M. et al., 2020). Therefore, cytokines were detected in piglets infected with PEDV at 3 days post-infection in this study.

Collectively, our results show that PEDV-infected Vero cells and IPEC-J2 expressed different cytokine profiles. The changes of some cytokines in ileum of piglets infected with PEDV were similar to those secreted by IPECJ2 cells infected with PEDV, and significantly different from those secreted by Vero cells. Our findings reveal that IPEC-J2 cells is an ideal cell model for PEDV studies *in vitro*, which will be very helpful to further study the interaction between PEDV infection and host innate immunity.

## Data availability statement

The original contributions presented in the study are included in the article/Supplementary material, further inquiries can be directed to the corresponding author.

## Ethics statement

This study was ethically approved by Hebei Agricultural University (China) Animal Welfare and Ethical Review Board (Permit Number: 1820026). The Hebei Agricultural University’s and China guidelines for the Care and Use of Laboratory Animals were followed.

## Author contributions

CY and LS contributed equally to this study by performing the experiments, analyzing data, and drafting the manuscript. LC performed animal infection, sample collection, and data analysis. LL, ZY, YW, and HG performed the experiment. TL prepared and provided the reagents exclusively for this research. QS conceived the study, performed animal experiments, and edited and finalized the manuscript. All authors contributed to the article and approved the submitted version.

## Funding

This work was supported by the National Natural Science Foundation of China (31372441 and 31772738). The study was also supported by the Research Funding of Hebei Agricultural University.

## Conflict of interest

The authors declare that the research was conducted in the absence of any commercial or financial relationships that could be construed as a potential conflict of interest.

## Publisher’s note

All claims expressed in this article are solely those of the authors and do not necessarily represent those of their affiliated organizations, or those of the publisher, the editors and the reviewers. Any product that may be evaluated in this article, or claim that may be made by its manufacturer, is not guaranteed or endorsed by the publisher.

## Supplementary material

SUPPLEMENTARY FIGURE S1 Polymerase chain reaction (PCR) detection of PEDV N gene in mucus of the small intestine.

## Abbreviations

PEDV: porcine epidemic diarrhea virus
PDCoV: porcine triangular coronavirus
TGEV: porcine transmissible gastroenteritis virus
SADS-CoV: porcine acute diarrhea syndrome coronavirus
PED: porcine epidemic diarrhea
ssRNA: single-stranded RNA
DMEM: Dulbecco’s Modified Eagle’s Medium
TCID_50_: tissue culture infectious dose
CPE: cytopathic effect
MOI: multiplicity of infection
BSA: bovine serum albumin
PBS: phosphate-buffered saline
ELISA: Enzyme-Linked Immunosorbent Assay
RoV: Rotavirus
dpi: days post infection

## References

[ref1] Annamalai T. Saif L. J. Lu Z. Jung K. (2015). Age-dependent variation in innate immune responses to porcine epidemic diarrhea virus infection in suckling versus weaned pigs. Vet. Immunol. Immunopathol. 168, 193–202. doi: 10.1016/j.vetimm.2015.09.006, PMID: 26433606 PMC7112776

[ref2] Arce C. Ramirez-Boo M. Lucena C. Garrido J. J. (2010). Innate immune activation of swine intestinal epithelial cell lines (IPEC-J2 and IPI-2I) in response to LPS from salmonella typhimurium. Comp. Immunol. Microbiol. Infect. Dis. 33, 161–174. doi: 10.1016/j.cimid.2008.08.003, PMID: 18799216

[ref3] Chauhan P. Nair A. Patidar A. Dandapat J. Sarkar A. Saha B. (2021). A primer on cytokines. Cytokine 145:155458. doi: 10.1016/j.cyto.2021.155458 33581983

[ref4] Chen J. Cui Y. Wang Z. Liu G. (2020). Identification and characterization of PEDV infection in rat crypt epithelial cells. Vet. Microbiol. 249:108848. doi: 10.1016/j.vetmic.2020.108848, PMID: 32979749 PMC7497550

[ref5] Chen Y. M. Helm E. T. Gabler N. Hostetter J. M. Burrough E. R. (2020). Alterations in intestinal innate mucosal immunity of weaned pigs during porcine epidemic diarrhea virus infection. Vet. Pathol. 57, 642–652. doi: 10.1177/0300985820932140, PMID: 32880235

[ref6] Chen Q. Li G. Stasko J. Thomas J. T. Stensland W. R. Pillatzki A. E.. (2014). Isolation and characterization of porcine epidemic diarrhea viruses associated with the 2013 disease outbreak among swine in the United States. J. Clin. Microbiol. 52, 234–243. doi: 10.1128/JCM.02820-13, PMID: 24197882 PMC3911415

[ref7] Chen P. Zhao X. Zhou S. Zhou T. Tan X. Wu X.. (2021). A virulent PEDV strain FJzz1 with genomic mutations and deletions at the high passage level was attenuated in piglets via serial passage in vitro. Virol. Sin. 36, 1052–1065. doi: 10.1007/s12250-021-00368-w, PMID: 33909220 PMC8080196

[ref8] Du J. Luo J. Yu J. Mao X. Luo Y. Zheng P.. (2019). Manipulation of intestinal antiviral innate immunity and immune evasion strategies of porcine epidemic diarrhea virus. Biomed. Res. Int. 2019, 1–9. doi: 10.1155/2019/1862531 PMC6874955 31781594

[ref9] Guo H. Yao Z. Chen L. Li L. Li Y. Wang Y.. (2020). Humoral immune responses in piglets experimentally infected with a field strain of porcine epidemic diarrhea virus. Vet. Microbiol. 246:108742. doi: 10.1016/j.vetmic.2020.108742, PMID: 32605747

[ref10] Hu Y. Xie X. Yang L. Wang A. (2021). A comprehensive view on the host factors and viral proteins associated with porcine epidemic diarrhea virus infection. Front. Microbiol. 12:762358. doi: 10.3389/fmicb.2021.762358, PMID: 34950116 PMC8688245

[ref11] Jung K. Hu H. Saif L. J. (2016). Porcine deltacoronavirus infection: etiology, cell culture for virus isolation and propagation, molecular epidemiology and pathogenesis. Virus Res. 226, 50–59. doi: 10.1016/j.virusres.2016.04.009, PMID: 27086031 PMC7114557

[ref12] Jung K. Miyazaki A. Saif L. J. (2018). Immunohistochemical detection of the vomiting-inducing monoamine neurotransmitter serotonin and enterochromaffin cells in the intestines of conventional or gnotobiotic (Gn) pigs infected with porcine epidemic diarrhea virus (PEDV) and serum cytokine responses of Gn pigs to acute PEDV infection. Res. Vet. Sci. 119, 99–108. doi: 10.1016/j.rvsc.2018.06.009, PMID: 29909130 PMC7111759

[ref13] Jung K. Saif L. J. (2015). Porcine epidemic diarrhea virus infection: etiology, epidemiology, pathogenesis and immunoprophylaxis. Vet. J. 204, 134–143. doi: 10.1016/j.tvjl.2015.02.017, PMID: 25841898 PMC7110711

[ref14] Koonpaew S. Teeravechyan S. Frantz P. N. Chailangkarn T. Jongkaewwattana A. (2019). PEDV and PDCoV pathogenesis: the interplay between host innate immune responses and porcine enteric coronaviruses. Front Vet Sci 6:34. doi: 10.3389/fvets.2019.00034, PMID: 30854373 PMC6395401

[ref15] Langel S. N. Paim F. C. Alhamo M. A. Buckley A. Van Geelen A. Lager K. M.. (2019). Stage of gestation at porcine epidemic diarrhea virus infection of pregnant swine impacts maternal immunity and lactogenic immune protection of neonatal suckling piglets. Front. Immunol. 10:727. doi: 10.3389/fimmu.2019.00727, PMID: 31068924 PMC6491507

[ref16] Lee C. (2016). Erratum to: porcine epidemic diarrhea virus: an emerging and re-emerging epizootic swine virus. Virol. J. 13:19. doi: 10.1186/s12985-016-0465-y, PMID: 26833094 PMC4736639

[ref17] Li W. Li H. Liu Y. Pan Y. Deng F. Song Y.. (2012). New variants of porcine epidemic diarrhea virus, China, 2011. Emerg. Infect. Dis. 18, 1350–1353. doi: 10.3201/eid1803.120002, PMID: 22840964 PMC3414035

[ref18] Lin H. Li B. Chen L. Ma Z. He K. Fan H. (2017). Differential protein analysis of IPEC-J2 cells infected with porcine epidemic diarrhea virus pandemic and classical strains elucidates the pathogenesis of infection. J. Proteome Res. 16, 2113–2120. doi: 10.1021/acs.jproteome.6b00957, PMID: 28506058

[ref19] Niu X. Wang Q. (2022). Prevention and control of porcine epidemic diarrhea: the development of recombination-resistant live attenuated vaccines. Viruses 14:1317. doi: 10.3390/v14061317, PMID: 35746788 PMC9227446

[ref20] Niu Z. Zhang Y. Kan Z. Ran L. Yan T. Xu S.. (2021). Decreased NHE3 activity in intestinal epithelial cells in TGEV and PEDV-induced piglet diarrhea. Vet. Microbiol. 263:109263. doi: 10.1016/j.vetmic.2021.109263, PMID: 34749283

[ref21] Pensaert M. B. de Bouck P. (1978). A new coronavirus-like particle associated with diarrhea in swine. Arch. Virol. 58, 243–247. doi: 10.1007/BF01317606, PMID: 83132 PMC7086830

[ref22] Song L. Chen J. Hao P. Jiang Y. Xu W. Li L.. (2022). Differential Transcriptomics analysis of IPEC-J2 cells single or Coinfected with porcine epidemic diarrhea virus and transmissible gastroenteritis virus. Front. Immunol. 13:844657. doi: 10.3389/fimmu.2022.844657, PMID: 35401515 PMC8989846

[ref23] Song D. Park B. (2012). Porcine epidemic diarrhoea virus: a comprehensive review of molecular epidemiology, diagnosis, and vaccines. Virus Genes 44, 167–175. doi: 10.1007/s11262-012-0713-1, PMID: 22270324 PMC7089188

[ref24] Stevenson G. W. Hoang H. Schwartz K. J. Burrough E. R. Sun D. Madson D.. (2013). Emergence of porcine epidemic diarrhea virus in the United States: clinical signs, lesions, and viral genomic sequences. J. Vet. Diagn. Investig. 25, 649–654. doi: 10.1177/1040638713501675, PMID: 23963154

[ref25] Sun R. Q. Cai R. J. Chen Y. Q. Liang P. S. Chen D. K. Song C. X. (2012). Outbreak of porcine epidemic diarrhea in suckling piglets, China. Emerg. Infect. Dis. 18, 161–163. doi: 10.3201/eid1801.111259, PMID: 22261231 PMC3381683

[ref26] Yu L. Dong J. Wang Y. Zhang P. Liu Y. Zhang L.. (2019). Porcine epidemic diarrhea virus nsp4 induces pro-inflammatory cytokine and chemokine expression inhibiting viral replication in vitro. Arch. Virol. 164, 1147–1157. doi: 10.1007/s00705-019-04176-2, PMID: 30799511

[ref27] Yuan C. Jin Y. Li Y. Zhang E. Zhang P. Yang Q. (2021). PEDV infection in neonatal piglets through the nasal cavity is mediated by subepithelial CD3(+) T cells. Vet. Res. 52:26. doi: 10.1186/s13567-020-00883-w, PMID: 33597007 PMC7888150

[ref28] Zakrzewski S. S. Richter J. F. Krug S. M. Jebautzke B. Lee I. F. Rieger J.. (2013). Improved cell line IPEC-J2, characterized as a model for porcine jejunal epithelium. PLoS One 8:e79643. doi: 10.1371/journal.pone.0079643, PMID: 24260272 PMC3829867

[ref29] Zhang H. Han F. Shu X. Li Q. Ding Q. Hao C.. (2022). Co-infection of porcine epidemic diarrhoea virus and porcine deltacoronavirus enhances the disease severity in piglets. Transbound. Emerg. Dis. 69, 1715–1726. doi: 10.1111/tbed.14144, PMID: 33960702

[ref30] Zhang Q. Yoo D. (2016). Immune evasion of porcine enteric coronaviruses and viral modulation of antiviral innate signaling. Virus Res. 226, 128–141. doi: 10.1016/j.virusres.2016.05.015, PMID: 27212682 PMC7111337

[ref31] Zhao X. Li Z. Zeng X. Zhang G. Niu J. Sun B.. (2017). Sequence analysis of the spike gene of porcine epidemic diarrhea virus isolated from South China during 2011-2015. J. Vet. Sci. 18, 237–243. doi: 10.4142/jvs.2017.18.2.237, PMID: 27515262 PMC5489471

