# Supplementary figures and images for: Differences in cytokines expression between Vero cells and IPEC-J2 cells infected with porcine epidemic diarrhea virus
Source: Front Microbiol. 2022 Nov 10;13:1002349. doi: 10.3389/fmicb.2022.1002349 (PMC9686284; doi:10.3389/fmicb.2022.1002349)

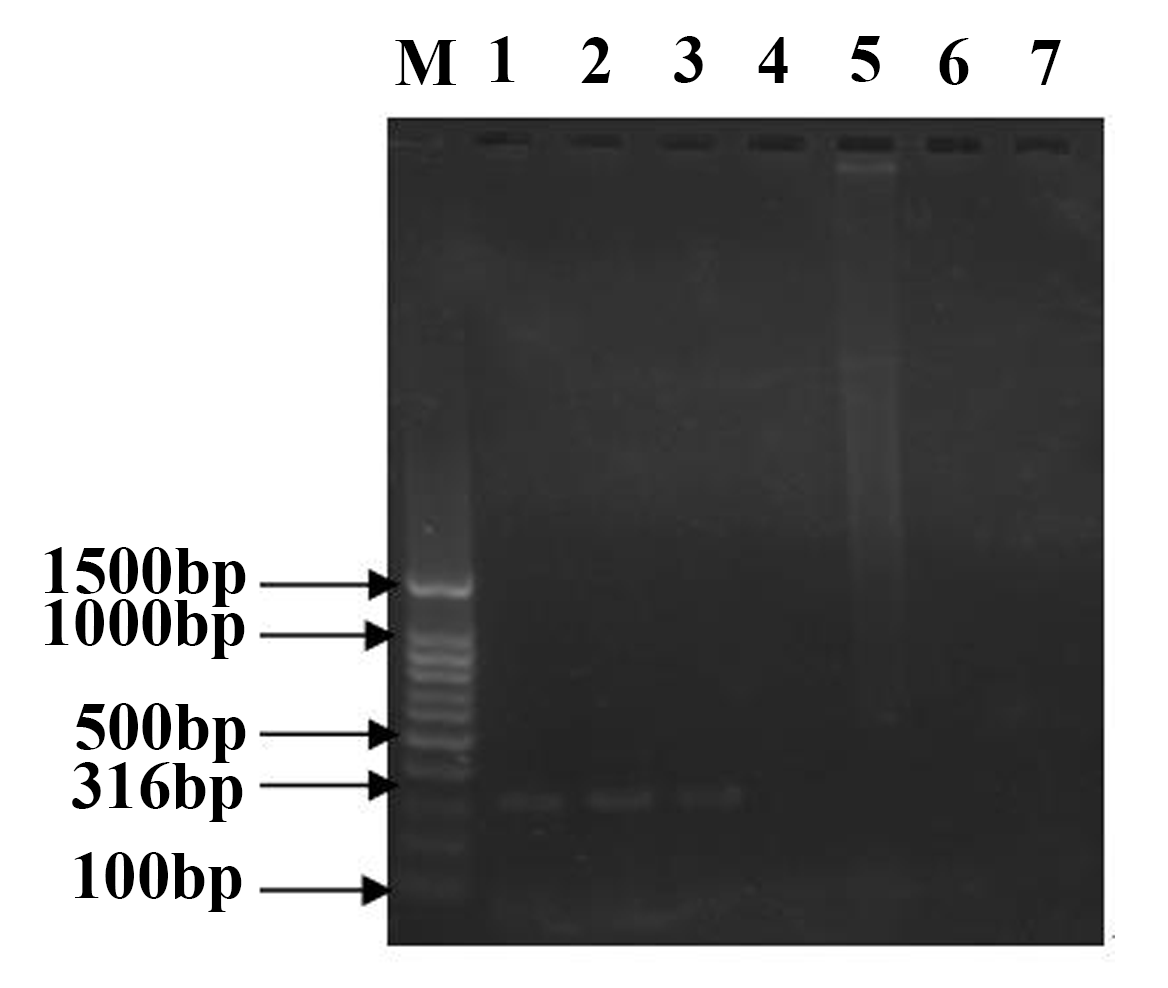

Supplement: SUPPLEMENTARY FIGURE S1 — Polymerase chain reaction (PCR) detection of PEDV N gene in mucus of the small intestine. [file Image_1.TIF]
